# Temporal landscape and translational regulation of A-to-I RNA editing in mouse retina development

**DOI:** 10.1186/s12915-024-01908-y

**Published:** 2024-05-07

**Authors:** Ludong Yang, Liang Yi, Jiaqi Yang, Rui Zhang, Zhi Xie, Hongwei Wang

**Affiliations:** 1 State Key Laboratory of Ophthalmology, Zhongshan Ophthalmic Center, Sun Yat-sen University, Guangdong Provincial Key Laboratory of Ophthalmology and Visual Science, Guangzhou, 510060 China; 2 MOE Key Laboratory of Gene Function and Regulation, Guangdong Province Key Laboratory of Pharmaceutical Functional Genes, State Key Laboratory of Biocontrol, School of Life Sciences, Sun Yat-sen University, Guangzhou, China

**Keywords:** A-to-I editing, Alternative splicing, Translation, Retina development

## Abstract

**Background:**

The significance of A-to-I RNA editing in nervous system development is widely recognized; however, its influence on retina development remains to be thoroughly understood.

**Results:**

In this study, we performed RNA sequencing and ribosome profiling experiments on developing mouse retinas to characterize the temporal landscape of A-to-I editing. Our findings revealed temporal changes in A-to-I editing, with distinct editing patterns observed across different developmental stages. Further analysis showed the interplay between A-to-I editing and alternative splicing, with A-to-I editing influencing splicing efficiency and the quantity of splicing events. A-to-I editing held the potential to enhance translation diversity, but this came at the expense of reduced translational efficiency. When coupled with splicing, it could produce a coordinated effect on gene translation.

**Conclusions:**

Overall, this study presents a temporally resolved atlas of A-to-I editing, connecting its changes with the impact on alternative splicing and gene translation in retina development.

**Supplementary Information:**

The online version contains supplementary material available at 10.1186/s12915-024-01908-y.

## Background

Adenosine-to-inosine (A-to-I) RNA editing, catalyzed by the ADAR (adenosine deaminases acting on RNA) family of enzymes, is the most prevalent type of RNA editing in metazoa. This process transforms adenosine into inosine in double-stranded RNA [1–3]. As a result of this editing, the ribosomes and splicing machinery interpret inosines as guanosines, leading to potentially non-synonymous substitutions if the editing occurs within the coding part of a transcript, thereby producing novel protein variants [4, 5]. Thus, A-to-I editing has a profound impact on targeted RNAs, not only altering their sequences compared to the genome but also influencing the cellular destiny of RNA molecules. To date, screening of the editome has revealed numerous A-to-I editing sites in various primate species [6, 7]. There is growing evidence that A-to-I editing plays a crucial role in nervous system development, performing various physiological functions such as regulating neuronal transmission, modulating synaptic plasticity, and controlling the timing of neurogenesis [8–11]. Nevertheless, given the increasing identification of A-to-I editing sites, the majority of them still have undefined spatial, temporal, and functional characteristics.

The survey of A-to-I editing in mammalian development not only unveils the dynamic nature of RNA editing but also sheds light on the intricate regulation of this process. While the brain has been the primary focus due to the high prevalence of A-to-I editing in the mammalian central nervous system (CNS) [12–14], the retina, being an extension and the most accessible part of the CNS, represents an ideal model for studying this phenomenon. As a multi-layered tissue crucial for photoreception and transduction of light stimuli, the retina is composed of three distinct layers: the outer nuclear layer (photoreceptors (rods and cones)), the inner nuclear layer (horizontal/bipolar/amacrine/Müller cells), and the ganglion cell layer (amacrine/ganglion cells) [15]. Proper developmental regulation of the retina is imperative for maintaining normal vision and ocular health, as abnormalities can lead to visual impairments and retinal pathologies. Several studies have highlighted the significant implications of A-to-I editing in the retina [16–19]. For instance, *Drosophila melanogaster* lacking the dADAR enzyme, which catalyzes A-to-I editing, exhibited structural abnormalities in the retina [20]. A-to-I editing of the *Gabra3* transcript in the chick retina is postulated to be important for the timing of excitatory-to-inhibitory GABA switch [17]. However, despite these findings, a comprehensive understanding of the prevalence, consequences, and significance of A-to-I editing in the developing retina remains limited.

A-to-I editing represents a fascinating layer of gene expression regulation that has been recognized for its role in illuminating the molecular basis of retina development. Numerous studies have shown that A-to-I editing plays a dynamic role in regulating transcriptome diversity and fine-tuning gene expression, including through interactions with alternative splicing [21–23]. However, the impact of A-to-I editing on translation diversity and translational regulation during retina development remains an underexplored area.

Herein, we delved into the temporal landscape and translational regulation of A-to-I editing during mouse retinal development, spanning five time points: E13, P0, P6, P21, and P42. These time points capture the progression of retina development, from proliferative retinal neural progenitors to retinal lamination and functional maturation of retinal cells [15]. Leveraging ultradeep transcriptomic data, we evaluated the A-to-I editing profiles of mouse retinas across these time points. Our analysis revealed distinct editing profiles at each time point, alongside discernible temporal patterns. Moreover, we explored the relationship between A-to-I editing and alternative splicing, revealing an interplay between these two processes. Furthermore, through ribosome profiling (Ribo-seq), we investigated the effect of A-to-I editing on gene translation. Our findings indicate that A-to-I editing acts as a buffer, diminishing the efficiency of translation, and produces coordinated effects when coupled with splicing events. In conclusion, our study presents a temporally resolved atlas of A-to-I RNA editing sites in the mouse retina, shedding light on the interplay between A-to-I editing and alternative splicing and their potential influence on the gene translation process.

## Results

### Characterization of high-confidence A-to-I editing sites across retina development

**Fig. 1:**
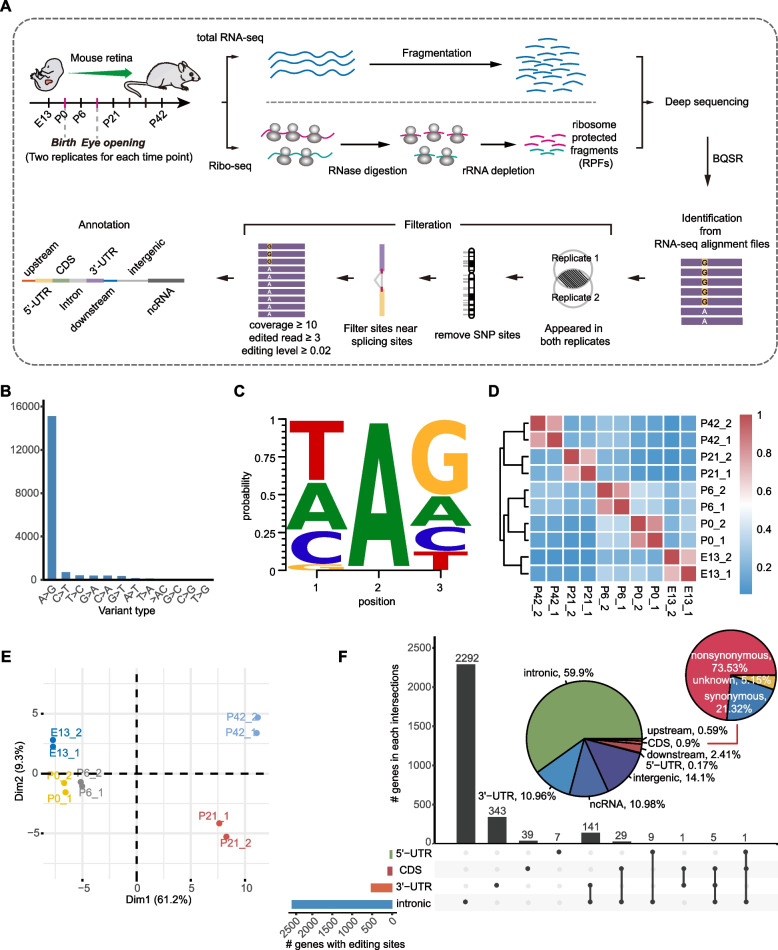
Genome-wide characterization of high-confidence A-to-I editing sites. **A** Schematic illustration of the experimental design and high-confidence A-to-I editing site identification and annotation. **B** Distribution of RNA editing types. The bar graph displays the number of each type of RNA editing, with “AG” representing A-to-I editing sites. **C** Nucleotide context around A-to-I editing sites, consistent with previous findings. **D** Heatmap of editing levels in different developmental time points. A value closer to 1 indicates a higher similarity of editing levels between samples. **E** Principal component analysis of editing levels in different developmental time points. **F** Distribution of editing sites on genomic regions. The inserted pie displays their relative fraction. Nonsynonymous refers to editing sites in the CDS that result in changes in amino acids, while synonymous refers to editing sites that do not cause such changes

To obtain a global landscape of A-to-I editing in the developing mouse retina, we performed total RNA-seq to profile five time points, including E13, P0, P6, P21, and P42. In total, the RNA-seq experiments yielded more than 1.19 billion raw reads, with an average of ~119 million reads per sample (Fig. 1A; Additional file 1: Supplementary Table 1). After quality control and data preprocessing, we applied REDItools2 [24] to characterize the RNA editing profiles. Our subsequent filtering steps, illustrated in Fig. 1A and Additional file 2: Fig. S1, resulted in a set of 17,874 high-confidence RNA editing sites, of which 15,109 were A-to-I editing sites, making up 84.53% of the entire set (Fig. 1B; Additional file 1: Supplementary Tables 2 and 3). This proportion is in line with prior research findings [25, 26]. Among these A-to-I editing sites, 8104 were previously reported by REDIportal, while the remaining 7005 were newly discovered, and both exhibited a similar motif pattern (Additional file 2: Fig. S2 and Fig. S3). Sanger sequencing of cDNA and gDNA further validated some newly discovered sites, including two sites specific to the mouse retina within *Rgs9bp*, thereby confirming their authenticity (see the “Methods” section; Additional file 2: Fig. S4). Moreover, the newly discovered sites were found to have a significant association with functions such as synapse and visual perception (Additional file 2: Fig. S3). At least, these results indicated that some, if not all, of these newly discovered sites are retina-specific. Given that A-to-I editing is the most prevalent type of editing and has significant impacts on development [14, 27, 28], we chose to focus our subsequent analysis solely on this type of editing.

We found that the level of guanosine was lower in the nucleotide before the editing site and higher in the nucleotide after, which aligns with the substrate requirements of ADAR editing (Fig. 1C). Hierarchical clustering analysis showed high consistency in editing levels between replicates and clear separation between developmental time points (Fig. 1D). The results of the principal component analysis reflected a developmental progression, from the embryonic (E13) to neonatal (P0 and P6) and then to eye-opening (P21 and P42), in line with the maturation of the retina (Fig. 1E). Consistent with prior studies [28–30], we found that the majority (59.9%) of A-to-I editing sites were presented within introns, with only a small proportion (0.9%) located within coding sequences (CDS). Among those within CDS regions, 73.53% led to non-synonymous changes (Fig. 1F). Overall, our analysis demonstrates the high reliability of the A-to-I editing sites we identified.

### Temporal changes of A-to-I editing across retina development

**Fig. 2:**
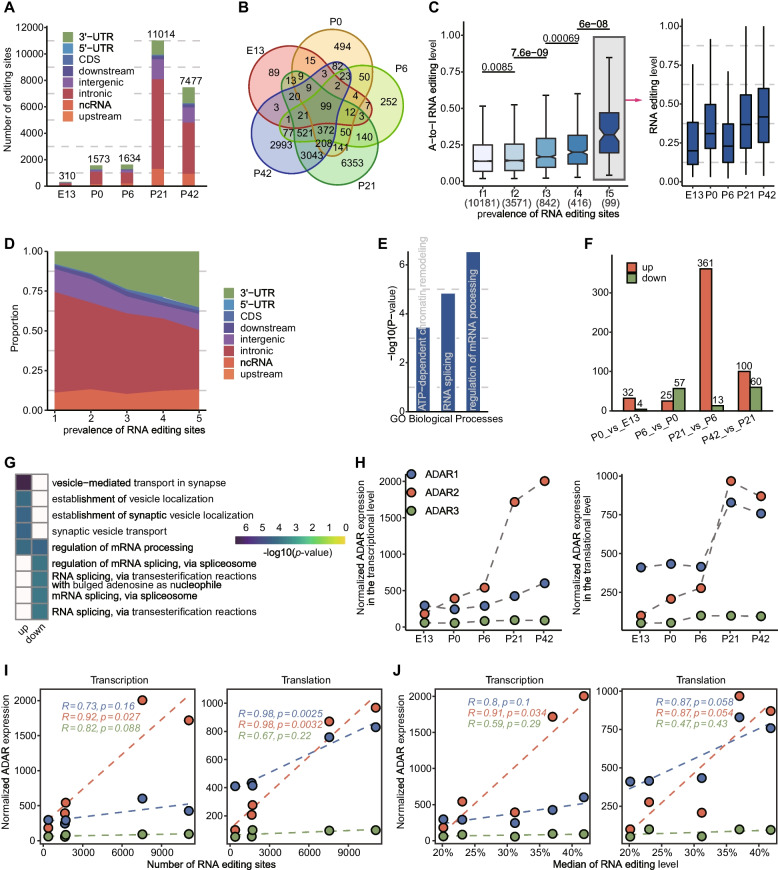
Temporal changes of A-to-I editing. **A** Temporal distribution of A-to-I editing sites. **B** Intersection of editing sites at each time point. **C** Editing levels grouped by prevalence. The “f1” refers to editing sites that appeared only once in the 5 time points, while “f5” refers to editing sites that are shared by all 5 time points. The editing levels of f5 in different developmental time points were shown on the right. Significance testing was performed by the Wilcoxon rank sum test, and *p*-values were shown at the top of the box plots. **D** Proportion of sites in different genomic regions concerning prevalence. It shows the proportion of sites in 3′-UTR increasing and the proportion of sites in intronic decreasing with increasing prevalence. **E** Enrichment analysis of editing genes shared by all developmental time points. The top-ranked enriched GO terms are shown here. **F** Bar plot showing the number of differential editing sites between adjacent developmental time points. **G** Enriched GO biological process terms of genes with differential up- and downregulated editing sites. **H** Normalized expression of three ADARs at the transcriptional (left) and translational level (right). **I** Pearson’s correlation analysis between the editing number of all sites and ADAR expression at the transcriptional and translational levels. **J** Pearson’s correlation analysis between editing levels of completely shared sites and ADAR expression at the transcriptional and translational levels, respectively

To investigate temporal dynamics of A-to-I editing, we initially quantified editing sites and noted substantial variation across time points, ranging from 310 to 11,014 sites (Fig. 2A). Despite this variability, a marked increase in site numbers was observed over time, with a steep surge occurring post eye-opening. Notably, this phenomenon could not be attributed to sequencing coverage bias, as no significant correlation between the number of A-to-I editing sites and sequencing depth was observed (Additional file 2: Fig. S5). We further categorized these editing sites into five groups based on their developmental prevalence. The majority (67.38%, 10,181 sites) were exclusive to a single time point, with only a negligible fraction (0.66%, 99 sites) shared across all time points (Fig. 2B). Analysis between different groups revealed substantial disparities in editing levels, with a trend of higher editing levels as prevalence increased (Fig. 2C). Intriguingly, shared editing sites displayed elevated editing activity that increased during retina development (Fig. 2C). These sites preferentially localized to 3′ UTR regions (Fig. 2D; Additional file 2: Fig. S6). Our enrichment analysis revealed that they were often located within genes associated with functions such as “regulation of mRNA processing,” “ATP-dependent chromatin remodeling,” and “RNA splicing” (Fig. 2E), suggesting a possible functional purpose for these sites. In contrast, timepoint-specific editing sites displayed relatively low levels of editing activity, which might be due to purifying selection that impedes their editing ability or prevalence [31].

The temporal changes of A-to-I editing were then studied by analyzing the differences in editing levels between adjacent time points using REDITs [32]. Our results showed that a total of 604 sites underwent differential editing, with a remarkable transformation in the number of differentially edited sites before and after eye-opening (Fig. 2F and Additional file 2: Fig. S7; Additional file 1: Supplementary Table 4). Our enrichment analysis indicated that as the retina developed, editing sites that experienced upregulation were primarily linked to synaptic vesicles, such as “vesicle−mediated transport in synapse” and “synaptic vesicle transport,” while those that were downregulated were mainly related to RNA splicing, such as “mRNA splicing, via spliceosome” and “regulation of mRNA splicing, via spliceosome” (Fig. 2G). These results suggest the involvement of A-to-I editing in retina development, particularly in modulating neurotransmitter release from synaptic vesicles and guiding alternative splicing decisions.

Furthermore, we investigated the relationship between ADAR expression and RNA editing. We found that ADAR2 had the most noticeable increase in expression throughout retina development, as seen at both transcriptional and translational levels, compared to the other two ADAR genes (Fig. 2H). Additionally, we found a significant positive relationship between the number of editing sites and ADAR2 expression, as indicated by Pearson’s correlation coefficients of 0.92 (*p*-value = 0.027) and 0.98 (*p*-value = 0.003) for transcription and translation, respectively (Fig. 2I). While no clear correlation was found between editing levels of all editing sites and ADAR1/2 expression, a marginally significant negative correlation emerged with the translational level of ADAR3 (Additional file 2: Fig. S8). Despite this, a portion of the editing variability could be attributed to ADAR expression, as indicated by a significant positive relationship between its transcription and editing levels of editing sites completely overlapped across all time points (Pearson’s *r* = 0.91, *p*-value = 0.034) and a marginally significant positive relationship between its translation and editing levels at the same sites (Pearson’s *r* = 0.87, *p*-value = 0.054) (Fig. 2J). Specifically, we found that the identified A-to-I editing sites covered approximately 46% of previously reported ADAR2 targets [12], but only 10% of ADAR1 targets (Additional file 2: Fig. S9). Collectively, our results suggest that ADAR2 may play a more important role without exclusive regulation by ADAR1/3 on RNA editing.

### Timepoint-specific A-to-I editing pattern on retina development

**Fig. 3:**
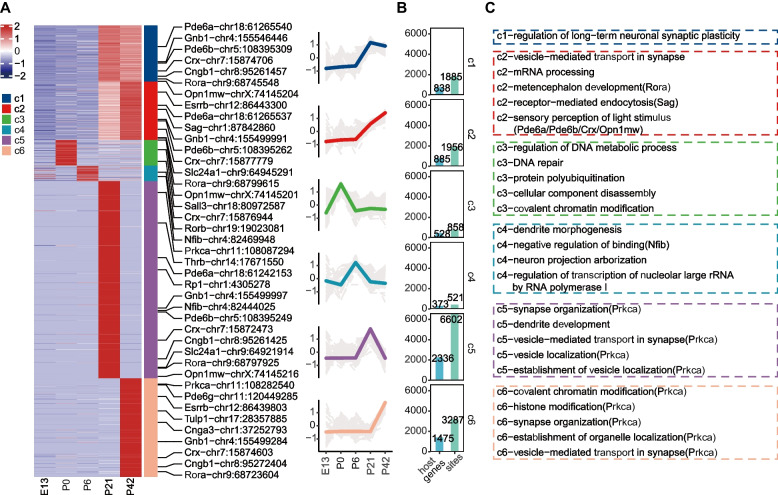
Developmental patterns of A-to-I editing. **A** Heatmap displaying the temporal distribution of A-to-I editing during development, with the right showing the distribution of retinal markers in these patterns. The line plot depicts the editing level trends for each pattern, with colored lines indicating the normalized mean editing level of each pattern, and gray lines representing the normalized editing level of an individual editing site. **B** Bar plots showing the number of genes and editing sites included in each editing pattern. **C** The top-ranked 5 or all enriched GO terms for each pattern in panel **A** are listed, with retina markers shown in parentheses after each function category

We next explored the RNA editome in greater detail to understand the changes in the editing pattern. By using mfuzz clustering [33], we identified six distinct groups of temporal editing profiles, as shown in Fig. 3A and Additional file 1: Supplementary Tables 5 and 6. The first cluster (c1), consisting of 1885 editing sites, showed a pattern of concurrent editing, with a sudden increase in editing levels following eye-opening. This pattern was also observed in cluster 2 (c2), which was made up of 1956 editing sites (Fig. 3B). Further analysis revealed that the sites within c1 and c2 were associated with functions such as “regulation of long-term neuronal synaptic plasticity” and “sensory perception of light stimulus” (Fig. 3C; Additional file 1: Supplementary Table 7). The editing patterns of clusters 3–6 were unique to their respective time points. Cluster 3 (c3), which was comprised of editing sites specific to P0, was characterized by functions related to those such as “regulation of DNA metabolic process,” “DNA repair,” and “covalent chromatin modification,” coinciding with actively cellular differentiation at this time point, marked by the formation of a substantial number of rod cells [34]. Interestingly, *Crx*, a crucial transcription for photoreceptor cell differentiation, was also edited during this time point. Cluster 4 (c4), made up of P6-specific editing sites, was characterized by functions related to those such as “neuron projection arborization” and “negative regulation of binding.” Cluster 5 (c5), made up of P21-specific editing sites, was characterized by functions related to those such as “synapse organization” and “vesicle−mediated transport in synapse,” and cluster 6 (c6), made up of P42-specific editing sites, was characterized by functions related to such as “covalent chromatin modification” and “histone modification,” suggesting that proper editing of sites in the retina may be necessary for functional maturation of retinal cells.

### Interplay between alternative splicing and A-to-I editing

**Fig. 4:**
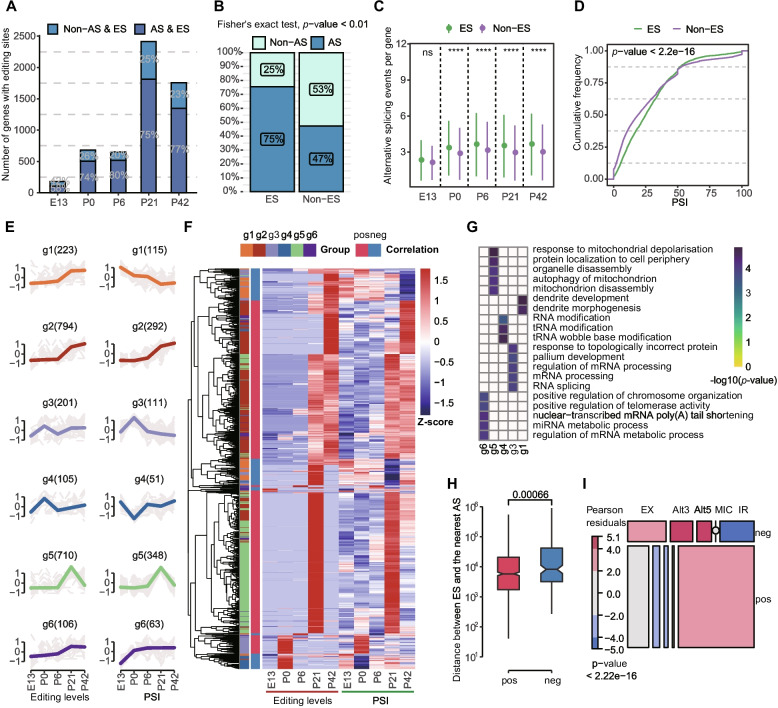
Interplay between A-to-I editing and alternative splicing. **A** Proportion and number of editing genes with and without alternative splicing in each developmental time point. **B** Association between A-to-I RNA editing and alternative splicing, by classifying genes into four categories based on the presence or absence of editing sites and alternative splicing events, and using Fisher’s exact test to determine significance (*p*-value < 0.01). **C** Comparison of the average number of splicing events between genes with and without editing. **D** Comparison of the percent spliced in (PSI) values between editing and non-editing genes. **E** Patterns of Normalized PSI values (left) and normalized editing levels (right) for strongly correlated pairs of splicing events and nearby editing sites. The colored line represents the overall trend of changes for each group, while each gray line reflects the change of a specific splicing or editing event. **F** Heatmap displaying the tendency of editing levels and splicing efficiency for strongly correlated pairs of splicing events and editing sites, with “pos” and “neg” indicating positive and negative correlation, respectively. **G** Heatmap displaying the top-ranked 5 or all enriched GO terms for six strongly correlated groups. **H** Comparison of the distances between paired positively correlated splicing events and editing sites with those of negatively correlated pairs. **I** Mosaic plot showing the distribution of different types of splicing events between positively and negatively correlated pairs of splicing events and editing sites. Significance testing was performed by Wilcoxon rank sum test: ns, not significant; ****p < 0.0001

To gain insights into the connection between RNA editing and alternative splicing, we examined their interrelation. By analyzing transcriptome data (see the “Methods” section; Additional file 1: Supplementary Table 8), we found that 73% of genes with RNA editing also exhibited alternative splicing (Fig. 4A). The presence of RNA editing was found to be significantly more prevalent among genes with splicing compared to those without, regardless of whether the transcript length was normalized (Fig. 4B; Additional file 2: Fig. S10; Fisher’s exact test, *p*-value < 0.01). When comparing genes with and without RNA editing but both with alternative splicing, we observed that edited genes had a greater number of alternative splicing events per gene (Fig. 4C) and higher splicing efficiency, quantified by the percent spliced in (PSI) value (Wilcoxon rank sum test, *p*-value < 2.2e−16) (Fig. 4D)*.* Our analysis also revealed a proximity between RNA editing and intron-retained (IR) events (Additional file 2: Fig. S11), suggesting that RNA editing may have a greater impact on IR events compared to other events, such as exon skipping and mutually exclusive exons (EX), alternative acceptors (Alt3), alternative donors (Alt5), and exon skipping for micro exons (MIC).

To further examine the extent of their developmental interrelation, we focused on analyzing 9390 pairs of editing sites (excluding those near the 4 nt intronic side of the splicing sites) and their corresponding nearby splicing events. We found that 2143 pairs were strongly correlated and categorized them into six distinct groups using Mfuzz (see the “Methods” section; Fig. 4E; Additional file 1: Supplementary Table 9). Our results showed that the changes in editing level and splicing efficiency followed a similar trajectory in groups 2, 3, 5, and 6, while groups 1 and 4 displayed a contrasting trajectory. Enrichment analysis showed that functions related to chromosome and mitochondrion, such as “positive regulation of chromosome organization” and “mitochondrion disassembly,” were over-represented in groups 5 and 6. This suggests that editing and splicing were closely intertwined in shaping message RNA. On the other hand, functions related to development and modification, such as “dendrite development” and “tRNA modification,” were over-represented in groups 1 and 4 (Fig. 4G). Correlation analysis between editing level and splicing efficiency showed that 957 pairs of editing sites and splicing events had a significantly strong relationship (absolute Pearson’s *r* ≥ 0.7 and *p*-value ≤ 0.05) (Fig. 4F). Positively correlated editing sites and splicing events were located closer together than negatively correlated ones (Wilcoxon rank sum test, *p*-value = 0.00066) (Fig. 4H and Fig. S7). IR events were more frequent than expected by chance in positive correlations, while EX events were dominant in negative correlations, suggesting that the impact of RNA editing may vary depending on the type of splicing event, with high editing activity tending to favor the preservation of nearby intron or the suppression of nearby exon (Fig. 4I).

### Alteration of translatome conferred by A-to-I editing

**Fig. 5:**
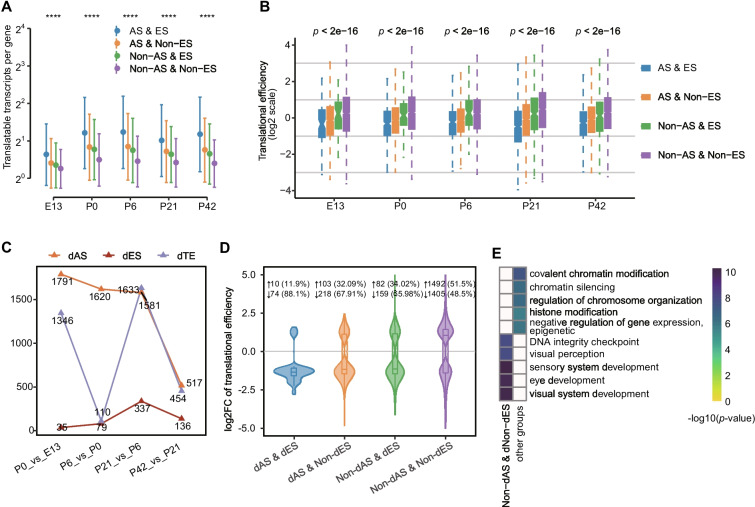
A-to-I editing induces changes in translatome. **A** Point-range plot displaying the mean number of translatable transcripts for four groups of genes (AS & ES, genes with both alternative splicing events and RNA editing sites; AS & Non-ES, genes with alternative splicing events but none editing sites; Non-AS & ES, genes without alternative splicing events but have editing sites; Non-AS & Non-ES, genes without either alternative splicing events or editing sites) classified based on the presence or absence of splicing events or editing sites. **B** Boxplot showing translational efficiency between different groups of genes (the groups classified as same as in **A**). **C** Number of genes with differential splicing events, differential editing sites, and differential translational efficiency in pairwise comparisons. **D** Violin plot comparing translational efficiency among four gene groups classified based on whether they exhibited differential editing or differential splicing efficiency. **E** Heatmap showing the top-ranked 5 enriched biological process GO terms for different gene groups in panel **D**. Significance testing was performed by Wilcoxon rank sum test: ****p < 0.0001

Translation rate and output can be impacted by RNA editing, but the extent of this impact is yet to be determined. To shed light on this, we used ribosome profiling to generate translation profiles, yielding an average of ~66 million raw reads per sample (see the “Methods” section; Additional file 1: Supplementary Table 1). Our results revealed that the combination of alternative splicing and RNA editing (AS & ES) resulted in the highest average number of actively translated transcripts per gene (Fig. 5A). This was followed by the group with only splicing (AS & Non-ES), then by the group with only editing (Non-AS & ES), and finally by the group with neither editing nor splicing (Non-AS & Non-ES). However, the Non-AS & Non-ES group had the highest translational efficiency, followed by the Non−AS & ES, AS & Non-ES, and AS & ES groups (Fig. 5B). These findings indicate that editing and splicing can increase coding capacity and diversify the translatome, with a synergistic effect when used together, for example, the editing level and splicing efficiency of retina-specific gene *Pcdh15*, *Stx3*, *Pde6b*, and *Tia1* synergistically inhibit their TE (Additional file 2: Fig. S12). Notably, the increasing diversity of translated transcripts was accompanied by heightened usage of RNA editing and splicing, with the latter having a more pronounced impact on gene’s translational efficiency than the former.

In light of these findings, we further examined the effect of RNA editing on gene translation. We performed differential translational efficiency (dTE) analysis between adjacent time points and found 2936 dTE genes that mirrored retina development, with two pronounced peaks in gene number between E13 and P0, and P6 and P21 (see the “Methods” section and Fig. 5C; Additional file 1: Supplementary Table 10). In parallel, we also found 3453 differential splicing efficiency (dPSI) genes and 440 differential editing level (dEL) genes (see the “Methods” section). When dTE genes were classified into four groups based on their dPSI or dEL status, we observed that in the Non-dAS & Non-dES group, there existed a close balance between up- and downregulated dTE genes, with 51.50% and 48.50%, respectively. The balance was disrupted in the presence of dPSI or dEL, resulting in the majority of dTE genes being downregulated in the Non-dAS & dES (65.98%), dAS & Non-dES (67.91%), and dAS & dES (88.1%) groups (Fig. 5D). These results suggest that RNA editing and splicing serve as a buffering mechanism to reduce gene translational efficiency, with both having a coordinated effect. Enrichment analysis further revealed that only the Non-dAS & Non-dES group had an over-representation of functions related to retina development, such as “visual perception” and “visual system development,” while the other three groups had an over-representation of functions related to the basic processes of life, such as “chromatin silencing” and “regulation of chromosome organization” (Fig. 5E).

## Discussion

ADAR-mediated A-to-I editing has been established as crucial for the normal development of organisms [26, 35, 36]. Disruptions to ADAR can lead to serious consequences such as locomotion and neuron defects seen in flies with mutant ADAR [37, 38]. However, the contribution of A-to-I editing to retina development has yet to be fully understood. We herein sought to understand the role of A-to-I editing in mouse retina development, with a specific focus on its effect on gene translation. By analyzing tens of thousands of editing events in mouse retina development, we created a detailed temporal map of the A-to-I editome, emphasizing the importance of A-to-I editing in regulating retina development*.*

A-to-I editing is catalyzed by ADARs, of which there are three members in mammals: ADAR1, ADAR2, and ADAR3. Our results suggest a more prominent role for ADAR2 in RNA editing during retina development, without exclusive regulation by ADAR1/3. However, the discordance between the transcription and translation levels of ADARs, particularly ADAR1, warrants further investigation. This discordance could arise from post-transcriptional regulatory mechanisms, such as RNA editing events that alter the coding sequence or stability of the ADAR transcripts themselves, as well as translational control mechanisms mediated by microRNAs, RNA-binding proteins, or structural features in the untranslated regions. The editing patterns produced by temporal changes in ADARs display specific and continuous characteristics, with the majority of editing sites exhibiting timepoint-specific changes during development, likely to meet the distinct demands of specialized retinal functions. Thus, the temporal pattern of A-to-I editing might facilitate the generation of cell types and the formation of functional neuronal circuitry.

The frequent observation of splicing in functions that were significantly enriched was noteworthy. This confirmed the close relationship between RNA editing and splicing, which was in agreement with prior findings that have emphasized the interplay between the two [21]. Our examination of alternative splicing events further showed that RNA editing may influence splice site selection. For instance, A-to-I editing has the ability to increase the number of splicing events by introducing potential donor or acceptor sites, and it could affect splicing efficiency by altering the structure and stability of sequences. While numerous examples in the literature support this, the rate of splicing may also influence the rate of RNA editing due to the availability of the exon complementary sequence necessary for dsRNA formation and ADAR recognition. It is clear that A-to-I editing and alternative splicing are interconnected, although the causality of RNA editing and alternative splicing is still debatable. Notably, RNA editing and splicing are both key pre-mRNA processing steps that can introduce substantial modifications to final gene products [21, 22]. Although the ability to dynamically regulate transcriptome diversity has been established, the potential influence of RNA editing and splicing on gene translation remains poorly understood. Our results indicate that A-to-I editing and splicing contribute to modifying gene translation. Specifically, A-to-I editing was found to have the potential to decrease translational efficiency through interaction with splicing. Potential mechanisms contributing to this phenomenon include the recoding of codons during editing, which leads to changes in amino acid identity and subsequently causes a deceleration of translation as ribosomes stall or pause at modified sites [39, 40]. Additionally, A-to-I editing might alter secondary/tertiary structures or subcellular localization of transcripts, thereby influencing the accessibility of the mRNA to ribosomes and other components of the translation machinery. Moreover, splicing might result in the removal of exons containing sequences crucial for translation regulation, such as upstream ORFs or binding sites of RNA-binding proteins. However, an in-depth understanding of the precise mechanisms through which these post-transcriptional modifications diminish the translation efficiency of transcripts associated with ribosomes requires further experimental investigation. To our knowledge, this study offers a pioneering depiction of the complex interplay between RNA editing, alternative splicing, and translation. However, the causal relationships between them need further experimental verification by manipulating the parameters such as transcription, RNA-processing, splicing, RNA editing, nuclear export, translation, and decay.

The current study provides substantial predictions and in silico confirmation. However, the identification of RNA editing sites is a challenging task. Although the screening process was designed to ensure the accuracy of the sites, it does not guarantee that every editing site is experimentally confirmed. Future studies focused on uncovering the relationship between RNA editing enzymes and splicing machineries will deepen our knowledge of retina development mechanisms. Furthermore, including a broader range of time points in the analysis, beyond the current focus on major phases of retina development, will provide a more comprehensive understanding of RNA editing and its role in nervous system development. Integrating with other technologies such as scRibo-seq will allow for a more in-depth analysis of RNA editing’s impact on cell type-specific translation and regulation.

## Conclusions

In summary, our investigation has yielded a comprehensive and highly credible atlas of A-to-I RNA editing sites in the developing mouse retina. Our results reveal the intercorrelation between A-to-I editing and alternative splicing. Ultimately, the interplay between A-to-I editing and alternative splicing holds the potential to enhance gene translation diversity, albeit with a trade-off in translational efficiency.

## Methods

### Tissue collection

Specimens of retinal tissue were obtained from C57BL/6 mice of wild-type origin, which were supplied by the Animal Centre of Southern Medical University in Guangzhou, China. To minimize individual variations, mice from the same litter and their offspring were used. Specifically, at embryonic day 13 (E13), retinas from 4 mice were used; at postnatal day 0 (P0), retinas from another set of 4 mice were used; and at P6, P21, and P42, retinas from 2 mice were used for each time point. The retinal tissue samples covered a diverse spectrum of developmental time points, encompassing E13, P0, P6, P21, and P42. After crushing in a tissue mashing machine (JXFSTPRP-24L, Shanghai Jingxin), the samples were immediately frozen in liquid nitrogen to preserve their quality. All animal experiments were approved by the Animal Ethics Committee of the Zhongshan Ophthalmic Center, Sun Yat-sen University (Guangzhou, China) with the permit number 2017-085A.

### Library preparation and sequencing

The total RNA-seq and Ribo-seq libraries for each sample were generated according to previously reported protocols [41]. In brief, the tissue samples were lysed using a mixture of mammalian lysis buffer (200 μl 5x Mammalian Polysome Buffer, 100 μl 10% Triton X-100, 10 μl DTT (100 mM), 10 μl DNase I (1U/μl, NEB, #M0303S), 2 μl Cycloheximide (50 mg/ml, Sigma–Aldrich, #C4859-1ML), 10 μl 10% NP-40, and 668 μl Nuclease-Free Water). After 20 min of incubation on ice, the lysates were clarified through centrifugation at 10,000×*g* for 3 min at 4 °C. The clarified lysates were then divided into 300-μl and 100-μl aliquots. The 300-μl aliquots were treated with 5 units of ARTseq Nuclease for 45 min at ambient temperature to perform nuclease digestion. The ribosome-protected fragments were purified using Sephacryl S-400 HR spin columns (GE Healthcare Life Sciences, #27-5140-01) and RNA Clean & Concentrator-25 kit (Zymo Research, #R1017). The ribosomal RNA was removed from the purified RNAs using the Ribo-Zero magnetic kit (Epicentre). The Ribo-seq library was constructed using the ARTseq^TM^ Ribosome Profiling Kit (Epicentre). The 100-μl aliquots were used for total RNA extraction and purification. The purified RNAs were linked to a 5′ adaptor, followed by reverse transcription and PCR amplification, culminating in a strand-specific total RNA-Seq library created using the VAHTSTM total RNA-Seq v2 Library Prep Kit from Illumina (Vazyme Biotech, #NR603). Notable, two biological replicates were sequenced for each developmental time point. The resulting libraries were then sequenced using the Illumina HiSeq 2500 following the manufacturer’s protocol, producing 2 × 125 bp paired-end reads for total RNA-seq and 1 × 51 bp single-end read runs for Ribo-seq.

### Data preprocessing

The raw read data of total RNA-seq and Ribo-seq were demultiplexed using CASAVA (v1.8.2), and then the 3′-end adapter was removed using Cutadapt (v1.8.1) [42]. To improve the quality of the data, low-quality sequences were trimmed using fastp with the following parameters: -q 30, -u 5 (v0.20.1) [43]. For the Ribo-seq data, an additional step was taken to filter the reads to retain only those with lengths between 25 and 35. To further clean the data, reads mapped to mouse rRNA and tRNA sequences were excluded. The remaining reads were then realigned to the mouse reference genome (GENCODE, GRCm38.p6) using HISAT2 (v2.1.0) [44]. Only the reads that were uniquely mapped reads were included in the downstream analysis. Finally, the number of reads for each gene was calculated using featureCounts (v1.6.4) [45].

### Identification and annotation of A-to-I editing sites

The total RNA-seq alignment BAM files were processed by removing PCR duplicates using Picard’s MarkDuplicates (v2.23.3). Base quality score recalibration was then conducted using the GATK BaseRecalibrator tool (v4.1.8.1) [46] to improve editing calling. Subsequently, RNA editing sites were detected using REDItools2 [24] with a parameter of -s 2. To ensure the accuracy of the editing sites, several steps were taken to minimize the risk of false positives, including (1) preserving sites that existed in both replicates, (2) trimming 12 nucleotides from the start and 2 nucleotides from the end of the reads, (3) eliminating sites located in SNPs [47, 48] and 4 nucleotides (nt) intronic side of the splicing site [12], (4) removing sites with multiple variant types, and (5) retaining sites with a minimum editing level of 0.02, at least 10 high-quality reads covered and at least 3 reads edited. The genomic features and amino acid changes of RNA editing sites were annotated using ANNOVAR (2020-06-07 release) [49]. Any sites with ambiguous annotations were excluded. The sequence context surrounding the RNA editing sites was analyzed using motifStack (v1.30.0) [50]. motifStack was utilized to analyze the sequence context surrounding the RNA editing sites. The above identification and annotation of A-to-I editing sites were integrated into a tailored workflow using Snakemake (v6.0.2) [51].

### Validation of A-to-I editing sites by Sanger sequencing

The A-to-I editing sites in the mouse retina were validated by Sanger sequencing, as detailed in the prior studies [52, 53]. Briefly, primers targeting the editing sites were designed, and the list of editing sites selected for validation, along with their corresponding primer sequences, is presented in Additional file 1: Supplementary Table 11. For RNA level validation, total RNA was extracted using EZ-press RNA Purification Kit (EZBioscience, #B0004D), followed by the reverse transcription with the HiScript III RT SuperMix for qPCR (Vazyme Biotech, #R323). For DNA level validation, genomic DNA was extracted using TIANamp Genomic DNA Kit (TIANGEN BIOTECH, #DP304). The synthesized cDNA and genomic DNA were then amplified using the 2 × Phanta Max Master Mix (Vazyme Biotech, #p515). The amplified products were subjected to direct sequencing on the ABI 3730xL DNA Analyzer by Guangzhou Tianyi Huiyuan Gene Technology Co., LTD.

### Characterization of editing pattern

Mfuzz (v2.46.0) [33] was used to study the editing patterns of sites during retina development. The following steps were taken: (1) a matrix that contained information on the editing level for each editing site in each time point was prepared, in order to create an ExpressionSet object, which is required by mfuzz; (2) mfuzz::mestimate was used to determine the optimal fuzzifier value (m); (3) the mfuzz function was employed with parameters c = 6 and m, determined in the previous step, to cluster editing sites; and finally, (4) the order of editing patterns was reordered based on the their peak timing during development.

### Estimation of gene expression and translational efficiency

The counts of all CDS from the 10 total RNA-seq and 10 Ribo-seq samples were consolidated into a single table, and genes with a total count of less than 10 across all samples were discarded. The DESeq2 (v1.26.0) [54] tool was used to estimate and remove the library size effect, and the gene expression was corrected based on their lengths to obtain the normalized gene expression. Finally, the translational efficiency was calculated by taking the ratio of the normalized gene expression at the translational level to the expression at the transcriptional level.

### Detection of differential editing sites

The REDIT-LLR function from the REDITs [32] software was used to identify differentially edited sites between adjacent developmental time points (P0 vs. E13, P6 vs. P0, P21 vs. P6, and P42 vs. P21). This was achieved by inputting a 2-row matrix that contained the number of edited and non-edited reads obtained from the output of REDItools2. Only sites with a *p*-value of 0.05 were considered statistically significant.

### Detection of differential translational efficiency

The DESeqDataSetFromMatrix function was used to identify differentially translational efficiency (dTE) genes between adjacent developmental time points, with a design formula of “library type + time + library type:time.” The input for this function was the combined counts obtained from the “Estimation of gene expression and translational efficiency” section. The “results” function was then used to extract dTE genes. Only genes with an absolute log2(fold change) of at least 1 and an adjusted *p*-value of 0.05, calculated using the Benjamini-Hochberg method, were considered statistically significant.

### Identification of alternative splicing events

Vast-tools (v2.5.1) [55] was used to identify alternative splicing (AS) events from total RNA-seq alignment BAM files. The parameters “--min_Fr 0.2 --noVLOM --p_IR” were applied to filter out events that did not have sufficient coverage (at least 25 reads for IR and 15 for others) in at least 20% of the samples and those that did not pass the binomial test. Notably, five distinct types of splicing events were revealed in this analysis, including exon skipping and mutually exclusive exons (EX), intron-retained (IR), alternative acceptors (Alt3), alternative donors (Alt5), and exon skipping for micro exons (MIC). The splicing efficiency, denoted as Percent Spliced In (PSI), for each alternative splicing event was determined using the Vast-tools algorithm. In essence, PSI was calculated as the ratio of inclusion reads to the sum of inclusion reads and exclusion reads.

### Detection of differential splicing events

The “compare” function of vast-tools was used to determine differential splicing events such as the PSI for AS events or percentage intron retained (PIR) for IR events, between adjacent developmental time points. The parameters “--min_dPSI 15,” “--min_range 5,” “--noVLOW,” “--p_IR,” and “-sp mm10” were employed.

### Correlation analysis between RNA editing and alternative splicing

To investigate the relationship between RNA editing and alternative splicing, each editing site and matched alternative splicing event was identified according to the following steps: (1) genes with both A-to-I editing and splicing events were selected; (2) for each editing site in these genes, the closest splicing event was assigned based on their coordinate information; and (3) Pearson correlation coefficient (*r*) was calculated for each editing site and matched splicing event based on changes in editing levels and PSI values during development. In cases where there are two editing sites associated with a specific splicing event, two correlations would be generated. Based on the correlation strength, the pairings were categorized into four groups: Strong (absolute value (*r*) ≥ 0.7), Moderate (0.7 > | *r* | ≥ 0.5), Weak (0.5 > | *r* | ≥ 0.3), and None (| *r* | < 0.3). Only pairs with a *p*-value of 0.05 for their correlation coefficient were considered as significantly correlated.

### Identification of actively translated transcripts

ORFquant (v1.1.0) [56] was used to identify actively translated transcripts from the Ribo-seq alignment BAM files, which adopts a greedy approach to determine the representative transcripts and estimate the impact of RNA editing on translation. Only transcripts that were deemed translatable in both replicates were kept for subsequent analysis. Here, GENCODE M23 was used for transcript annotation.

### Enrichment analysis

ClusterProfiler (v3.14.3) [57] was used to perform Gene Ontology (GO) biological process enrichment analysis, with an adjusted *p*-value of 0.05, calculated using the Benjamini-Hochberg method, being taken into account. To reduce the redundancy of enriched terms, a simplification process was implemented based on the hierarchical relationships between similar GO terms.

### Supplementary Information

Additional file 1: Supplementary Table 1. Summary of the number of reads for total RNA-seq and Ribo-seq data in mouse retina development. Supplementary Table 2. Summary of identified A-to-I editing sites in the developing mouse retina. Note that ‘strand’ represents the strand where the site is located, with 0 for the reverse strand and 1 for the forward strand. Supplementary Table 3. Depths of A-to-I sites across all replicates. Supplementary Table 4. Summary of differential editing sites between adjacent time points. Note that ‘DE level’ represents the absolute change of the editing level between the two time points, as stated in column3; ‘*p*-value’ represents the statistical measure by REDITs to evaluate the significance of the change in editing levels; and ‘dRES’ represents the direction of the change in editing levels. Supplementary Table 5. Clustering information of developmental editing patterns. Note that ‘Membership’ represents the fuzzy membership degree of editing sites belonging to the class. Supplementary Table 6. Clustering information of sites in retinal markers. Supplementary Table 7. Summary of the GO enrichment analysis of genes for different developmental editing patterns. Note that ‘geneID’ represents the gene symbols being analyzed presented in the GO term. Supplementary Table 8. Summary of identified alternative splicing events in the developing mouse retina. Supplementary Table 9. Correlation between editing sites and splicing events. Note that ‘dis.min’ represents the distance between the editing site and its paired splicing event. Supplementary Table 10. Summary of differential translational efficiency (TE) genes. Note that ‘lfcSE’ represents the standard error of the log2FoldChange estimate. Supplementary Table 11. List of editing sites selected for validation.

Additional file 2: Fig. S1 Proportion of A-to-I editing sites among all variants after filtering through various steps. Fig. S2 The Venn diagram shows the number of editing sites included in REDIportal, and the bar plot shows the top 10 (ranked by *p-*value) biological processes (BPs) enriched by sites not included in REDIportal. Fig. S3 Comparison of nucleotide context around Known and Novel A-to-I editing sites. Fig. S4 Validation of A-to-I editing sites by Sanger sequencing. Fig. S5 The Spearman’s correlation between the number of editing sites and the number of uniquely mapped reads. Fig. S6 Actual number of A-to-I editing sites changes during retina development. Fig. S7 UpSet plot showing the number and overlap of differential editing sites between adjacent time points. Fig. S8 Association analysis between ADAR expression and A-to-I editing. (A) Translational efficiencies of ADAR genes in different time points. (B) Pearson’s correlation between the editing levels of A-to-I RNA editing sites and the expression of ADARs at the transcriptional and translational level, respectively. Fig. S9 The Venn diagram shows the number of editing sites targeted by ADAR1 and ADAR2 in mouse retina. Fig. S10 Association between A-to-I RNA editing and alternative splicing following transcript length normalization. Fisher’s exact test to determine significance (*p*-value < 0.01). Fig. S11 Distance between the A-to-I editing site and their paired splicing event. Fig. S12 The heatmap shows that the editing level (EL) and PSI value of retina-specific genes undergo co-directional changes during development, thereby collectively suppressing their translational efficiency (TE).

## Data Availability

All data generated or analyzed during this study are included in this published article, its supplementary information files, and publicly available repositories. The total RNA-seq and Ribo-seq sequencing data is available in the NCBI Gene Expression Omnibus (GEO) with the accession number GSE104884.

## Abbreviations

A-to-I: Adenosine to inosine
ADAR: Adenosine deaminases acting on RNA
AS: Alternative splicing
Alt3: Alternative acceptors
Alt5: Alternative donors
CDS: Coding sequences
CNS: Central nervous system
dEL: Differential editing level
dPSI: Differential splicing efficiency
dTE: Differentially translational efficiency
ES: Editing sites
EX: Exon skipping and mutually exclusive exons
IR: Intron-retained
MIC: Exon skipping for micro exons
PSI: Percent spliced in
