# Supplementary material for: Temporal landscape and translational regulation of A-to-I RNA editing in mouse retina development
Source: BMC Biol. 2024 May 7;22:106. doi: 10.1186/s12915-024-01908-y (PMC11077751; doi:10.1186/s12915-024-01908-y)
Supplement: Supplementary file 2 — Additional file 2: Fig. S1 Proportion of A-to-I editing sites among all variants after filtering through various steps. Fig. S2 The Venn diagram shows the number of editing sites included in REDIportal, and the bar plot shows the top 10 (ranked by p-value) biological processes (BPs) enriched by sites not included in REDIportal. Fig. S3 Comparison of nucleotide context around Known and Novel A-to-I editing sites. Fig. S4 Validation of A-to-I editing sites by Sanger sequencing. Fig. S5 The Spearman’s correlation between the number of editing sites and the number of uniquely mapped reads. Fig. S6 Actual number of A-to-I editing sites changes during retina development. Fig. S7 UpSet plot showing the number and overlap of differential editing sites between adjacent time points. Fig. S8 Association analysis between ADAR expression and A-to-I editing. (A) Translational efficiencies of ADAR genes in different time points. (B) Pearson’s correlation between the editing levels of A-to-I RNA editing sites and the expression of ADARs at the transcriptional and translational level, respectively. Fig. S9 The Venn diagram shows the number of editing sites targeted by ADAR1 and ADAR2 in mouse retina. Fig. S10 Association between A-to-I RNA editing and alternative splicing following transcript length normalization. Fisher’s exact test to determine significance (p-value < 0.01). Fig. S11 Distance between the A-to-I editing site and their paired splicing event. Fig. S12 The heatmap shows that the editing level (EL) and PSI value of retina-specific genes undergo co-directional changes during development, thereby collectively suppressing their translational efficiency (TE). [file 12915_2024_1908_MOESM2_ESM.pdf]

Supplementary Materials for

Temporal Landscape and Translational Regulation of A-to-I RNA Editing in Mouse Retina Development

Ludong Yang[1,#], Liang Yi[1,#], Jiaqi Yang[1], Rui Zhang[2], Zhi Xie[1,*], Hongwei Wang[1,*]

This file includes:

Supplementary Figures S1-S12

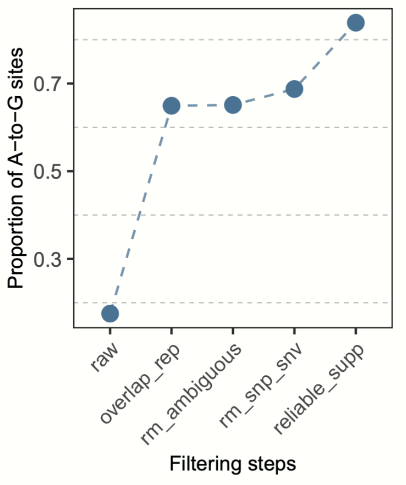

Fig. S1 Proportion of A-to-I editing sites among all variants after filtering through various steps. Note that 'raw' represents all editing sites identified by REDITools2; 'overlap_rep' represents editing sites occurred in both replicates; 'rm_ambigouous' indicates the remaining sites after removing the sites with multiple variant types; 'rm_snp_snv' represents the remaining sites after removing sites located at SNPs and SNVs; and 'reliable_supp' represents the retention of editing sites with a minimum editing level of 0.02, at least 10 high-quality reads coverage, and a minimum of 3 edited reads.

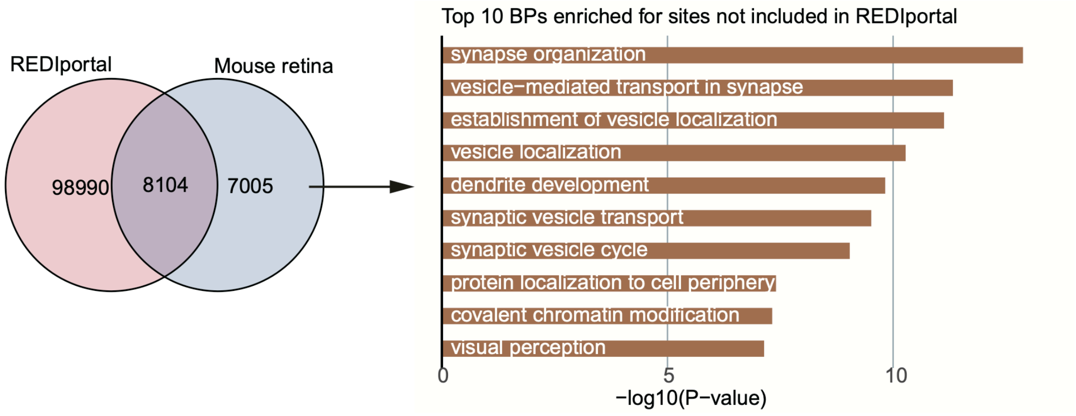

Fig. S2 The Venn diagram shows the number of editing sites included in REDIportal, and the bar plot shows the top 10 (ranked by *p*-value) biological processes (BPs) enriched by sites not included in REDIportal.

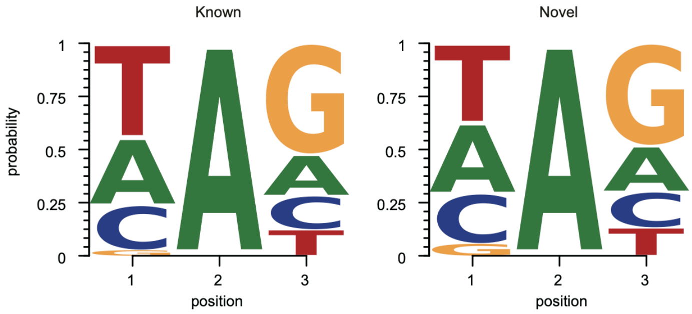

Fig. S3 Comparison of nucleotide context around Known and Novel A-to-I editing sites. Known sites are annotated in REDIportal, while Novel sites are unannotated in REDIportal.

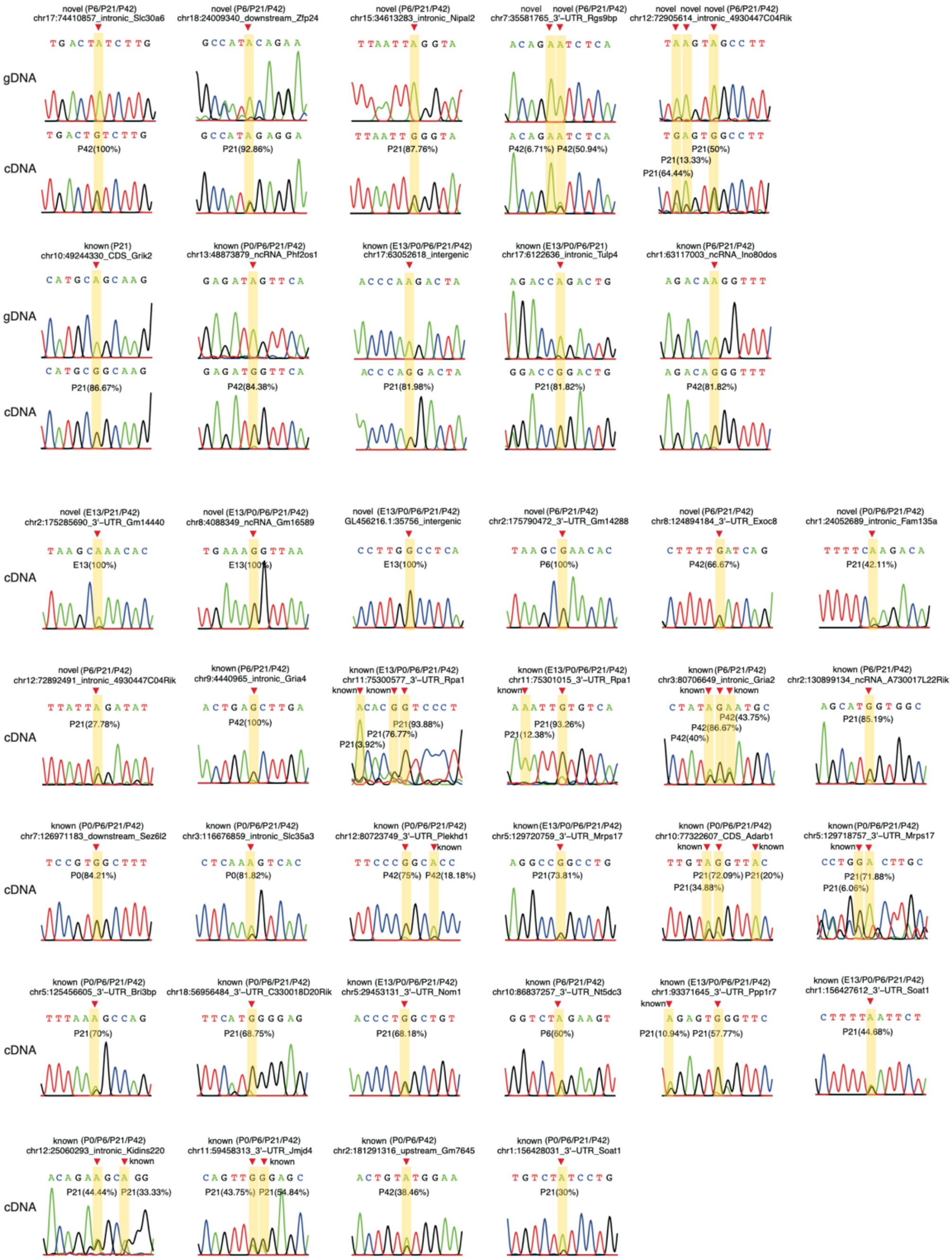

Fig. S4 Validation of A-to-I editing sites by Sanger sequencing. The source cDNAs were from mouse retinas. “known” refers to sites annotated in REDIportal, while “novel” indicates those newly discovered in this study. The sites were marked with red triangles. Specifically, the samples where these sites were observed are indicated in the figure using the format: novel (P6/P21/P42). Additionally, the source samples utilized for cDNA/gDNA Sanger sequencing and the predicted editing rates are labeled in the figure using the format: P21 (27.78%). Furthermore, we have appended all this information, including the corresponding primer sequences, to Additional file 1: Supplementary Table 11.

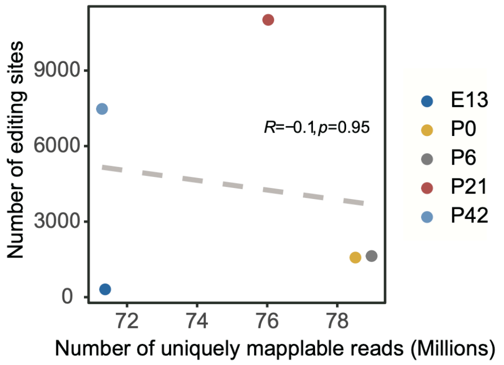

Fig. S5 The Spearman's correlation between the number of editing sites and the number of uniquely mapped reads.

Feature	E13	P0	P6	P21	P42
intronic	171	923	814	6787	3871
3'-UTR	60	325	366	1084	1214
ncRNA	38	142	184	1231	882
intergenic	30	116	181	1509	1141
downstream	3	33	48	234	232
5'-UTR	1	3	5	16	15
CDS	6	26	28	92	78
upstream	1	5	8	61	44

Fig. S6 Actual number of A-to-I editing sites changes during retina development.

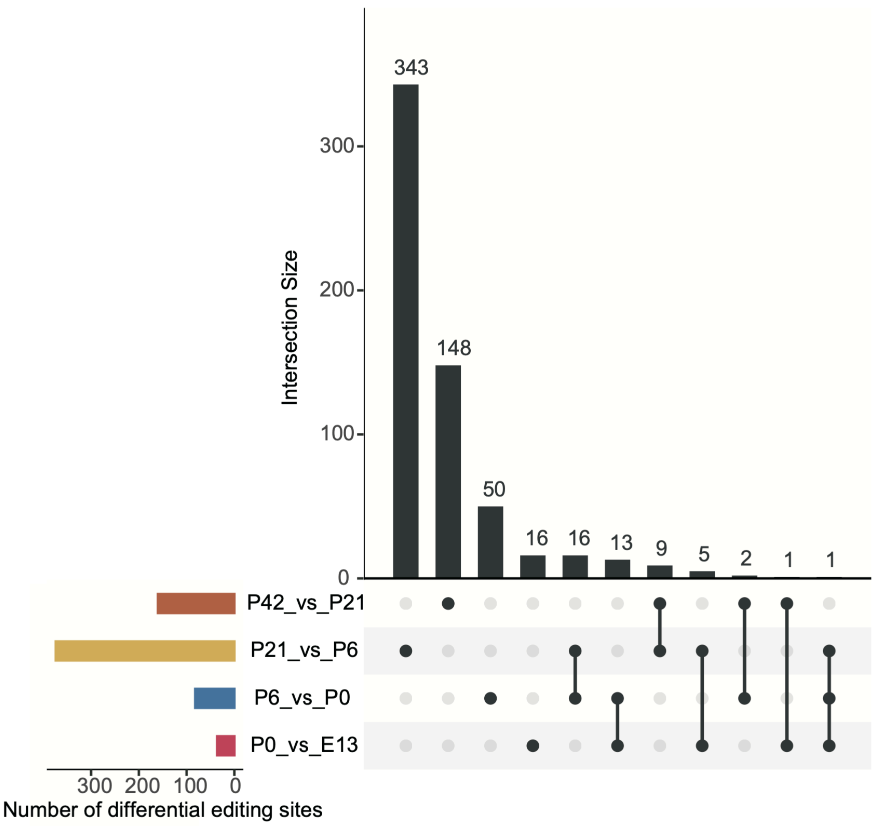

Fig. S7 UpSet plot showing the number and overlap of differential editing sites between adjacent time points.

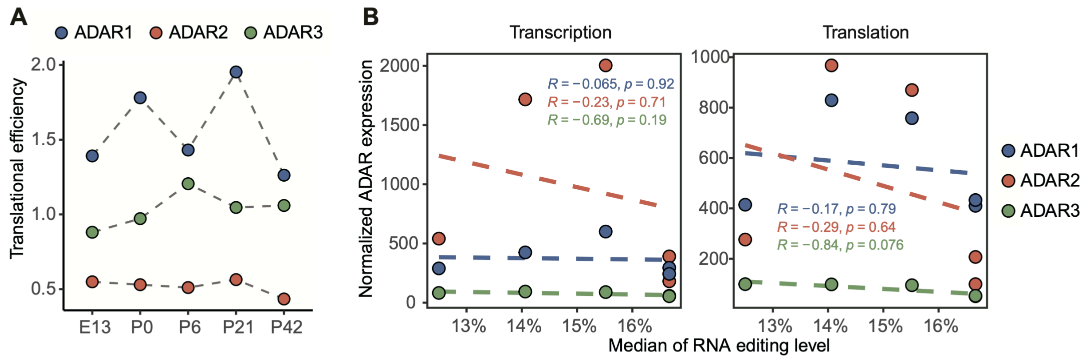

Fig. S8 Association analysis between ADAR expression and A-to-I editing. (**A**) Translational efficiencies of ADAR genes in different development stages. (**B**) Pearson's correlation between the editing levels of A-to-I RNA editing sites and the expression of ADARs at the transcriptional and translational level, respectively.

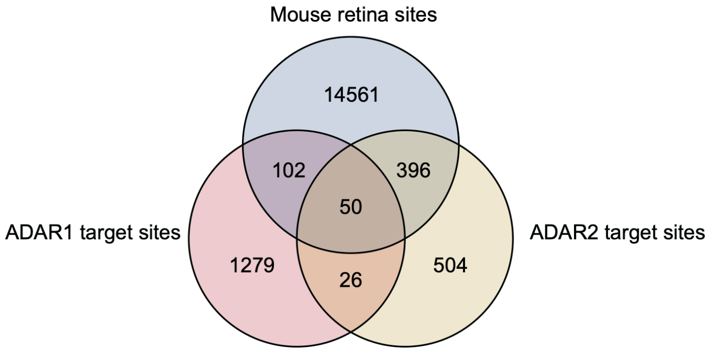

Fig. S9 The Venn diagram shows the number of editing sites targeted by ADAR1 and ADAR2 in mouse retina.

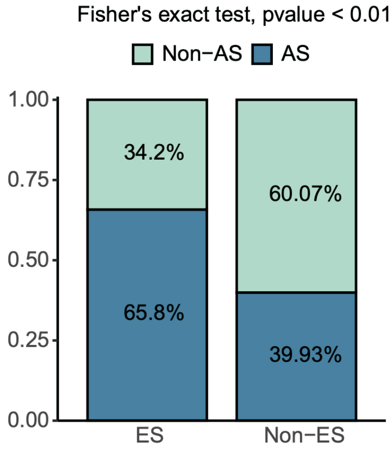

Fig. S10 Association between A-to-I RNA editing and alternative splicing following transcript length normalization. Fisher's exact test to determine significance (*p*-value < 0.01).

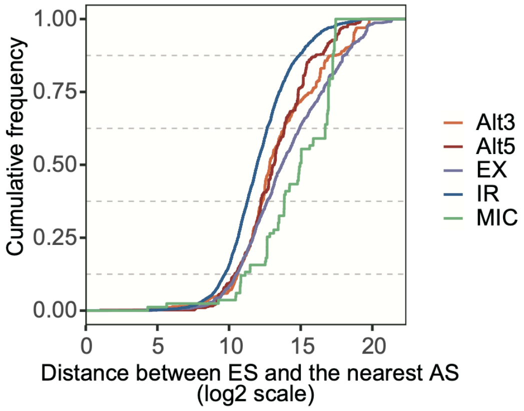

Fig. S11 Distance between the A-to-I editing site and their paired splicing event.

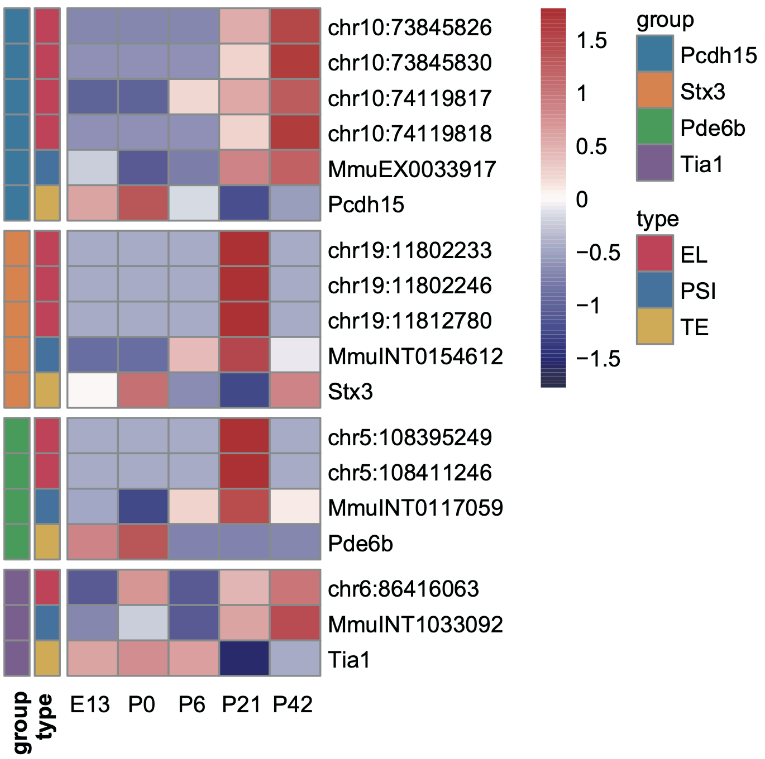

Fig. S12 The heatmap shows that the editing level (EL) and PSI value of retina-specific genes undergo co-directional changes during development, thereby collectively suppressing their translational efficiency (TE).
